# Gene and mutation independent therapy via CRISPR-Cas9 mediated cellular reprogramming in rod photoreceptors

**DOI:** 10.1038/cr.2017.57

**Published:** 2017-04-21

**Authors:** Jie Zhu, Chang Ming, Xin Fu, Yaou Duan, Duc Anh Hoang, Jeffrey Rutgard, Runze Zhang, Wenqiu Wang, Rui Hou, Daniel Zhang, Edward Zhang, Charlotte Zhang, Xiaoke Hao, Wenjun Xiong, Kang Zhang

**Affiliations:** 1 Guangzhou Women and Children's Medical Center, Guangzhou Medical University, Guangzhou 510623, China; 2 Shiley Eye Institute, Institute for Engineering in Medicine, Center for Genetic Therapy at Institute for Genomic Medicine, University of California, San Diego, La Jolla, California 92093, USA; 3 Department of Biomedical Sciences, City University of Hong Kong, Hong Kong SAR, China; 4 Guangzhou KangRui Biological Pharmaceutical Technology Company, Guangzhou 510005, China; 5 Department of Clinical Laboratory Medicine, Xijing Hospital, Fourth Military Medical University, Xi'an, Shanxi 710032, China; 6 Veterans Administration Healthcare System, San Diego, California 92037, USA

## Dear Editor,

We report a gene therapy strategy using CRISPR/Cas9-mediated cellular reprogramming by switching a mutation-venerable/sensitive cell type to a mutation-insensitive/resistant cell type, therefore restoring tissue architecture and function. We applied this strategy to retinitis pigmentosa (RP), a major cause of blindness characterized by retinal rod photoreceptor degeneration caused by numerous mutations in many genes. By reprogramming rod to cone-like photoreceptors *in situ* by inactivating *Nrl* or *Nr2e3*, we show an increase in cone-like cells with remarkable concomitant preservation of both cone and rod photoreceptors and retinal tissue, with restoration of visual function. Our approach demonstrates the feasibility of cellular reprogramming in preventing degeneration and preserving tissue and function, and points to a novel approach in treating human diseases in a gene and mutation independent manner.

Gene therapy shows great promise in treating many human diseases, yet a major drawback of the current technology is that it can only be directed to a particular mutation or a single gene at best. This makes it difficult to apply gene therapy to a spectrum of diseases and a broad patient population. Similarly, repair and regeneration of tissues using endogenous stem cells represents an important goal in regenerative medicine and promising efforts have been demonstrated in mouse liver^1^, zebra fish heart^2^, and human lens^3^. However, the starting cells used to regenerate tissues must possess normal genetic makeup and function, which represents a challenge in many cases of human disease. One approach to overcome the above drawbacks in tissue repair and regeneration is therapeutic cellular reprogramming, which switches a cell type sensitive to a mutation to a functionally related cell type that is resistant to the same mutation. Therefore, this strategy would render the underlying mutation irrelevant, with consequent preservation of tissue architecture and function.

We tested this hypothesis on RP, a retinal rod photoreceptor-specific disease. RP is one of the most common degenerative diseases of the eye, affecting over one million patients worldwide. RP is characterized by primary rod photoreceptor death and degeneration, followed by secondary cone death^4^. It can be caused by numerous mutations in over 200 genes, which limits the therapeutic impact of conventional gene therapy strategies. Acute gene knockout of rod determinant *Nrl* was shown to reprogram adult rods into cone-like cells, rendering them resistant to effects of mutations in RP-specific genes and consequently preventing secondary cone loss^5^. *Nrl* acts as a master switch gene between rods and cones and activates a key downstream transcription factor *Nr2e3*. *Nrl* and *Nr2e3* function in concert to activate a rod-specific gene transcription network and control rod differentiation and fate^6^. Loss of function in either *Nrl* or *Nr2e3* reprograms rods to a cone cell fate.

**Figure 1:**
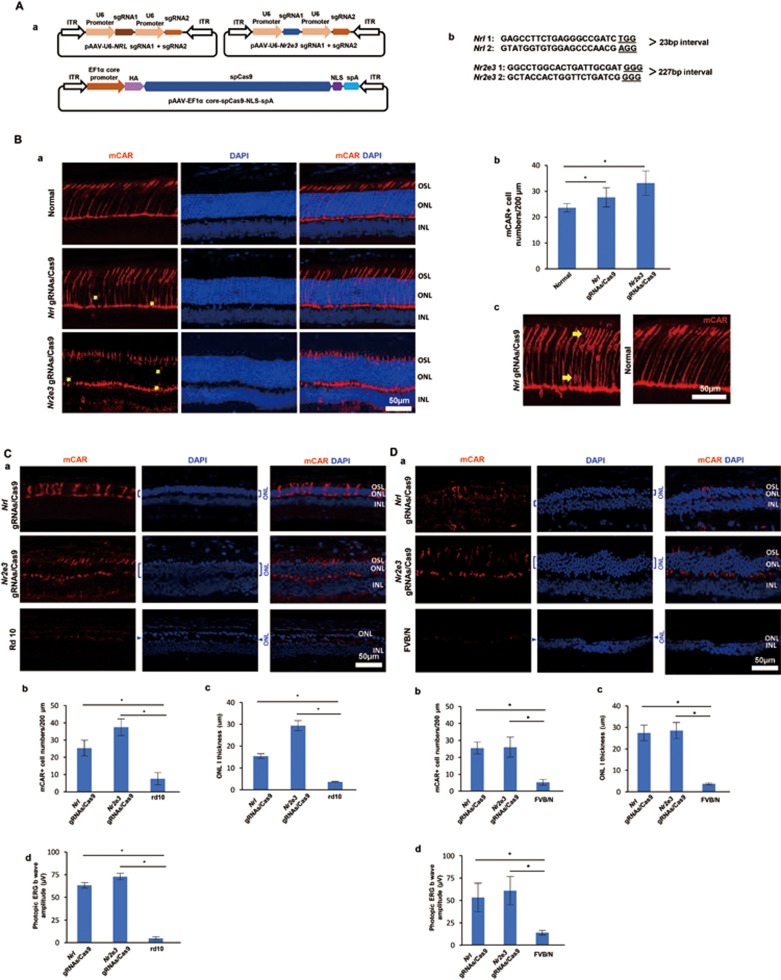
**(A)** AAV vector construction. a. Schematic of AAV vector construction for gRNAs and spCas9 to target *Nrl* and *Nr2e3* in mouse retina. ITR, inverted terminal repeats; EF1α, elongation factor 1-alpha; HA, human influenza hemagglutinin; NLS, nuclear localization signal; spA, short polyA. b. List of target sequences for *Nrl* and *Nr2e3* knockdown. PAM sequences were underlined. **(B)** More cone-like cells were observed in WT mouse retinas after transduction with AAV-gRNAs/Cas9. a. Immunofluorescence analysis of mCAR^+^ cells in mouse retina. Arrows point to possible ectopic mCAR^+^ cones. OSL, outer segment layer; ONL, outer nuclear layer; INL, inner nuclear layer. b. Increase of total mCAR^+^ cells in AAV-gRNAs/Cas9-treated eyes (^*^P < 0.05, student's *t*-test, *n* = 6). mCAR-positive cells within whole ONL were counted. Three adjacent sections from one retina were counted to get an average number of mCAR^+^ cells in each sample. c. Zoomed image showed that an mCAR^+^ cell, with nucleus at the lower ONL area, has a normal cone outer segment. **(C)** CRISPR/Cas9 knockdown strategy rescued retinal photoreceptor degeneration in rd10 mice. a. Immunofluorescence analysis of mCAR^+^ cells in rd10 mouse retina treated with AAV-gRNAs/Cas9. rd10 mice were treated at P7 and analyzed at P60. b. Quantification of total mCAR^+^ cells in retina treated with AAV-gRNAs/Cas9 (^*^P < 0.05, Student's *t*-test, *n* = 8). mCAR-positive cells within whole ONL were counted. Three adjacent sections from one retina were counted to get an average number of mCAR^+^ cells in each sample. c. Quantification of ONL thickness showed increased ONL thickness (^*^P < 0.05, Student's *t*-test, *n* = 8). d. Quantification of b wave amplitude in AAV-gRNAs/Cas9-treated, and uninjected mice. rd10 mice were injected at P7 and tested at P50 (^*^P < 0.05, Student's *t*-test, *n* = 6). **(D)** CRISPR/Cas9 knockdown strategy rescued retinal photoreceptor degeneration in FVB/N mice. a. Immunofluorescence analysis of mCAR in FVB/N mouse retina treated with AAV-gRNAs/Cas9. FVB/N mice were treated at P7 and analyzed at P50. b. Quantification of total mCAR^+^ cells in retina treated with AAV-gRNAs/Cas9 (^*^P < 0.05, Student's *t*-test, *n* = 6). mCAR-positive cells within whole ONL were counted. Three adjacent sections from one retina were counted to get an average number of mCAR^+^ cells in each sample. c. Quantification of ONL thickness showed increased ONL thickness (^*^P < 0.05, Student's *t*-test, *n* = 6). d. Quantification of b wave amplitude in AAV-gRNAs/Cas9-treated, and uninjected mice. FVB/N mice were injected at P7 and tested at P50 (^*^P < 0.05, Student's *t*-test, *n* = 6).

To test a CRISPR/Cas9-based cellular reprogramming strategy to treat RP, we employed two AAV vectors, one expressing Cas9, and another carrying gRNAs targeting the *Nrl* or *Nr2e3* gene (Figure 1A a, b). To assess if simultaneously targeting two sites by two gRNAs in the same gene has a higher targeting and inactivation efficiency than that by a single gRNA, we designed constructs that have either one or two gRNAs targeting *Nrl* or *Nr2e3*. We tested gene mutagenesis rates in mouse fibroblasts using a T7E1 nuclease assay, which cuts a mismatched double stranded DNA template. The results showed that the two-gRNA system had a much higher editing efficiency than that of a single-guide RNA (Supplementary information, Figure S1A a, b). We therefore adopted a double gRNA targeting strategy in all subsequent *in vivo* experiments.

We next delivered the two gRNAs/Cas9 constructs using AAV vectors to normal mice via subretinal injection at postnatal day 7 (P7) and sacrificed mice for histology at P30. Retinas were frozen-sectioned and stained for cone markers, including cone arrestin (mCAR) and short wavelength opsin (S-opsin)^7^. We observed a reprogrammed photoreceptor phenotype with two gRNAs *in vivo.* In normal retinas, cone nuclei reside at the top layer of outer nuclear layer (ONL), while rod nuclei fill the rest of ONL (Figure 1B a). We observed there were many mCAR^+^ cells in the lower ONL areas in retinas treated with *Nrl* gRNAs/Cas9 or *Nr2e3* gRNAs/Cas9 (Figure 1B a, b). The extra mCAR^+^ cells at the lower ONL areas have a normal outer segment (Figure 1B c). Consistent with activation of a cone-like gene expression program, these mCAR^+^ cells were also positive for another cone-specific marker S-opsin (Supplementary information, Figure S1B). We used quantitative RT-PCR (qRT-PCR) to measure the relative expression levels of rod or cone genes in reprogrammed retinas and controls. As expected, there was down-regulation of rod-specific genes with concomitant upregulation of cone-specific genes (Supplementary information, Figure S1C).

Next, to explore the possibility of using the CRISPR/Cas9-based strategy for gene therapy in RP, we targeted *Nrl* or *Nr2e3* in two different RP animal models. Rd10 mice represent a common model for autosomal recessive RP in humans with rod photoreceptor degeneration^8^. We injected AAV-gRNAs/Cas9 into the subretinal space of rd10 mice at P7. To determine the effect of AAV-gRNAs/Cas9 treatment, electroretinography (ERG) responses were tested in eyes of live mice to measure the electrical activity of cone photoreceptors (photopic response) at P60. All eyes treated with AAV-gRNAs/Cas9 exhibited significantly improved photopic b-wave values, suggesting improved cone function (Figure 1C d, Supplementary information, Figure S2C). Scotopic ERG result showed no change of a-wave, while there was a small but significant increase of scotopic b-wave amplitude at higher light intensity levels (Supplementary information, Figure S2A-S2B). Consistent with the visual function improvement, histology analysis of the AAV-gRNAs/Cas9-treated-rd10 retinas showed preservation of a number of mCAR^+^ cells (Figure 1C a, b). In addition, AAV-gRNAs/Cas9 treatment led to significantly improved preservation of the ONL thickness compared with non-injected control as well as preservation of cells positive for S-opsin and PNA staining (Figure 1C c, Supplementary information, Figure S1D).

The FVB/N mouse strain provides another model of rapid photoreceptor degeneration^9^, which is caused by another mutation in *PDE*. We injected AAV-gRNAs/Cas9 into the subretinal space at P7, and tested ERG responses at P50, followed by histology analysis. Consistent with results from rd10 mice, histology analysis of the AAV-gRNAs/Cas9-treated FVB/N retinas showed greater preservation of mCAR^+^ cells and significantly improved preservation of ONL thickness (Figure 1D a-c). The eyes treated with AAV-gRNAs/Cas9 also exhibited significantly improved photopic b-wave values, suggesting improved cone function (Figure 1D d).

In this study, we showed that targeted inactivation of *Nrl* or *Nr2e3* via CRISPR/Cas9-mediated genome editing can reprogram rod to cone-like photoreceptors, rescue retinal rod and cone degeneration and restore visual function in two different mouse RP models. In addition, our double gRNA strategy towards a single gene significantly increased targeting efficiency. In RP, cone photoreceptor death with consequent loss of day light and color vision function has the most severe impact on patient quality of life, yet it appears to be secondary to rod death. Our cellular reprogramming approach significantly rescues and restores both rod and cone function. This reprogramming strategy is especially attractive as the natural course of the disease inevitably results in loss of both rods and cones at an advanced stage, which eventually leads to legal blindness.

Our approach shows promise of cellular reprogramming in preventing degeneration and restoring tissue function, and points to a novel approach in treating human diseases in a gene and mutation independent manner.

Note added in proof: an independent study with similar results has been published recently in *Nat Commun*^10^.

## Supplementary Information

Supplementary information, Figure S1 **A**. AAV vector genome-editing efficiency.
