# Supplementary material for: Gene and mutation independent therapy via CRISPR-Cas9 mediated cellular reprogramming in rod photoreceptors
Source: Cell Res. 2017 Apr 21;27(6):830–3. doi: 10.1038/cr.2017.57 (PMC5518875; doi:10.1038/cr.2017.57)
Supplement: Supplementary information, Figure S1 — A. AAV vector genome-editing efficiency. [file cr201757x1.pdf]

Supplemental Figure 1

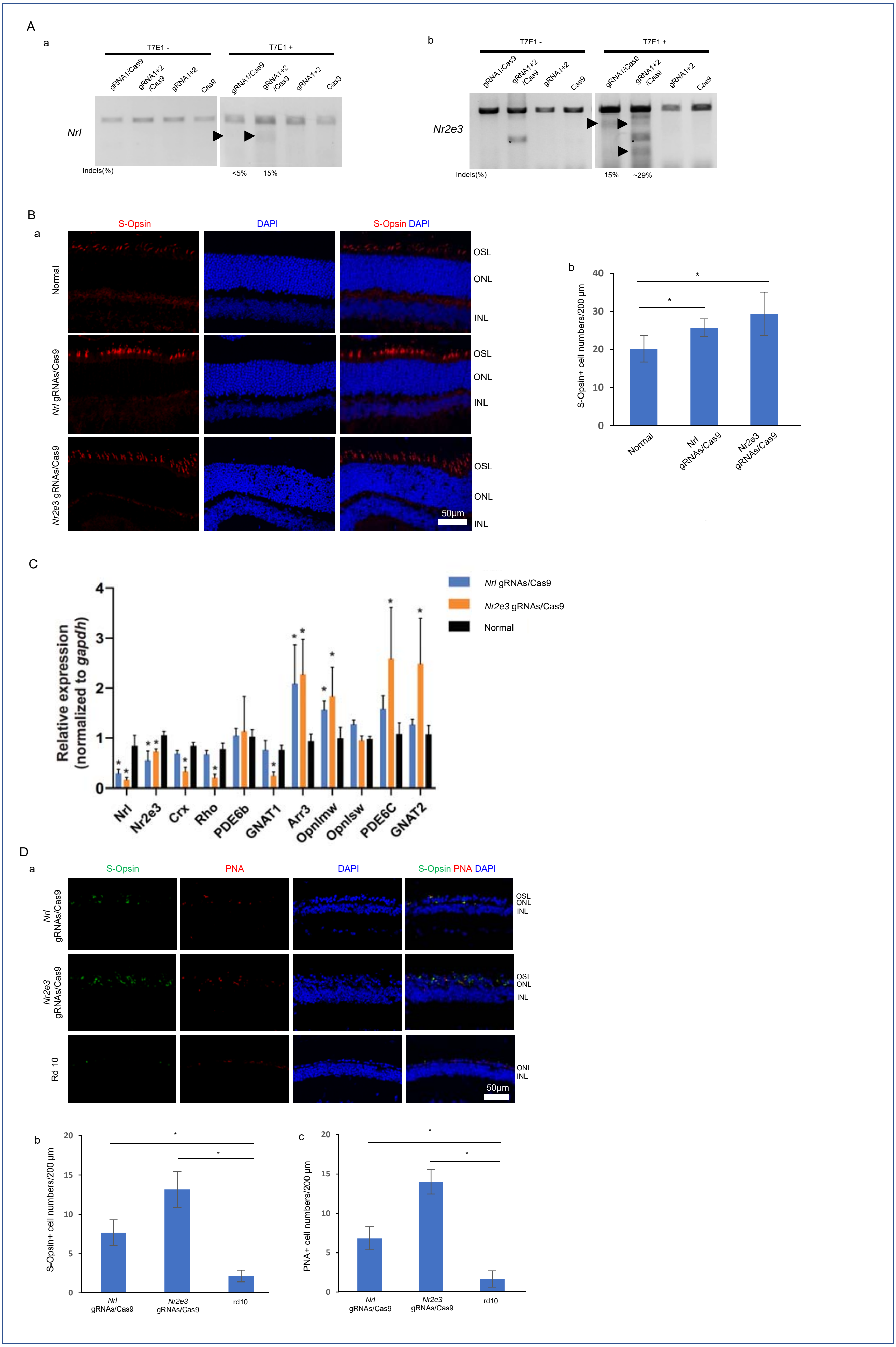

Supplemental Figure 2

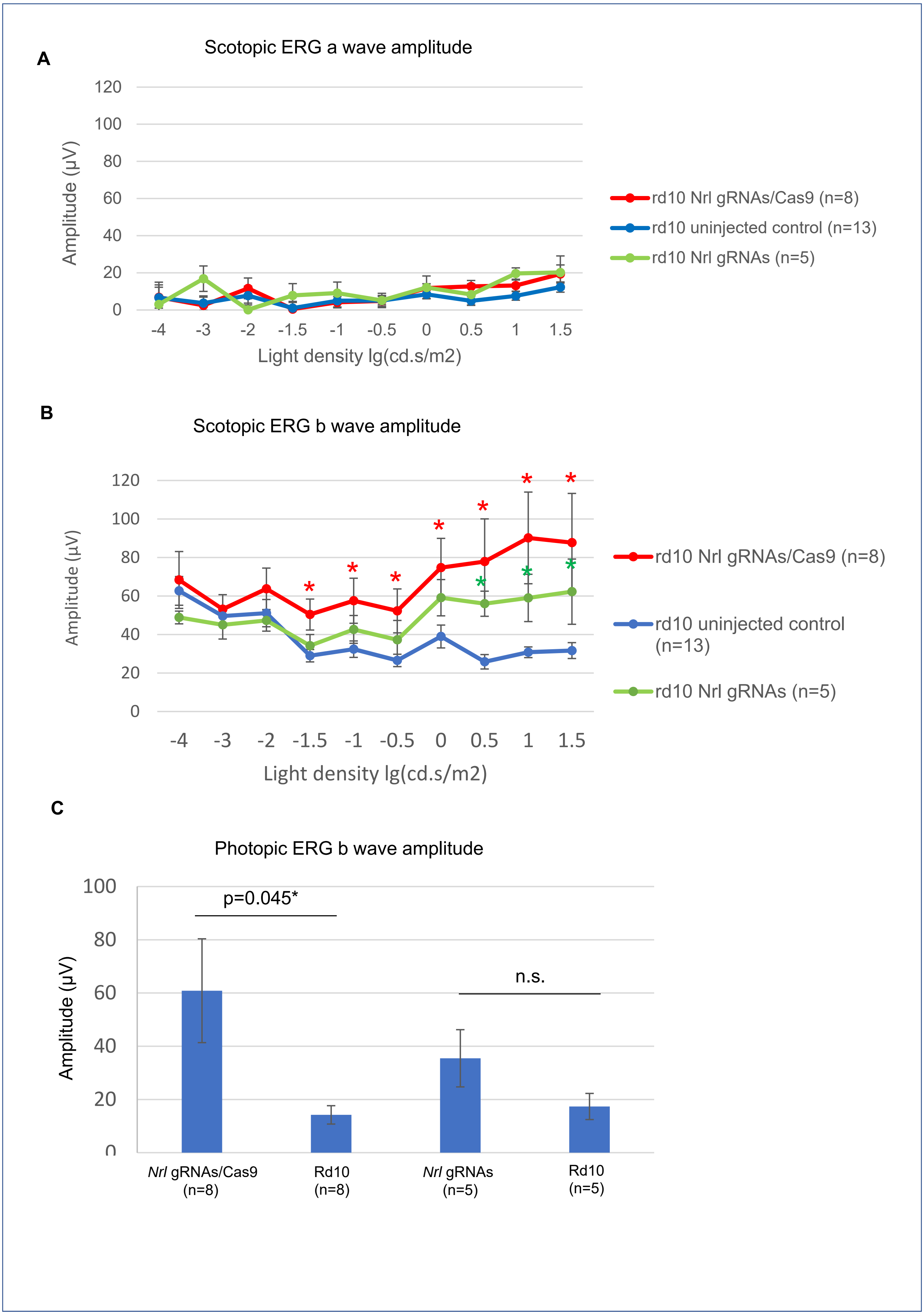

Supplementary information, Figure S1 A. AAV vector genome-editing efficiency. a. and b. T7E1 assay of *Nrl* and *Nr2e3* gRNAs in mouse embryonic fibroblasts (MEFs). MEFs were co-transfected with *Nrl* or *NR2e3* vectors together with Cas9 vector, and T7E1 assay was carried out using genomic DNA. Vectors with single gRNA were included as controls. The black arrows indicate cleaved DNA produced by T7E1 enzyme that is specific to heteroduplex DNA caused by genome editing. Dual gRNA has better efficiency (28% for *Nrl*, 50% for *Nr2e3*) than single gRNA (<10% for *Nrl*, 27.3% for *Nr2e3*). Asterisks indicate shorter PCR amplicons mediated by dual gRNAs/Cas9 cutting. The mutation frequency was calculated from the proportion of cut band intensity to total band intensity. **B.** More S-Opsin+ cells in WT mouse retinas after transduction with AAV-gRNAs/Cas9. a. Immunofluorescence analysis showed normal retina injected with AAV-gRNAs/Cas9 had extra S-Opsin$^+$ cells. b. Increased S-Opsin$^+$ cells in AAV-gRNAs/Cas9 treated eyes (* P<0.05, student's t-test, n=6). S-Opsin positive outer segments were counted. Three adjacent sections from one retina were counted to get an average number of S-Opsin$^+$ outer segment in each sample. **C.** RT-qPCR analysis of rod specific genes (*Nrl, Nr2e3, Crx, Rho,* and *GNAT1*) and cone specific genes (*Arr3, M-Opsin, S-Opsin, PDE6C,* and *GNAT2*) in wild-type retina treated with AAV-*Nrl* gRNAs/Cas9 or AAV-*Nr2e3* gRNAs/Cas9. RNA from each group were extracted form whole retina tissue. Results are shown as mean ± s.e.m. (*P <0.05. Student's *t*-test, n≥3). **D.** CRISPR/Cas9 knockdown strategy rescues retinal photoreceptor degeneration in rd10. a. Immunofluorescence analysis of S-Opsin and PNA in rd10 mouse retinas treated with AAV- gRNAs/Cas9. b. quantification of total S-Opsin$^+$ outer segments and PNA labeled cells in rd10 retina treated with AAV-gRNAs/Cas9 (*P<0.05, student's *t*-test, n=6).

Supplementary information, Figure S2 A, B. Scotopic ERG results of rd10 mice retreated with *Nrl* gRNAs/Cas9, *Nrl* gRNAs alone, or uninjected. Results are shown as mean ± s.e.m. (*P <0.05. Student's t-test). **C.** Quantification of b wave amplitude in AAV-*Nrl* gRNAs/Cas9 injected, AAV-*Nrl* gRNAs alone injected, and uninjected mice. Results are shown as mean ± s.e.m. (*P <0.05. Paired student's *t*-test).

Supplementary information, Materials and Methods

Plasmids

To construct dual gRNA expression vectors, pAAV-U6 gRNA-EF1a mCherry[11] was used. Both 20bp target sequences were sub-cloned into the vector separately as described previously[11]. The second gRNA together with U6 promoter was then amplified and inserted into the first gRNA expression vector. The CRISPR/Cas9 target sequences (20 bp target and 3 bp PAM sequence showed with underline) used in this study are shown as following: GAGCCTTCTGAGGGCCGATC TGG, and

GTATGGTGTGGAGCCCAACG AGG for *Nrl* knockdown, GGCCTGGCACTGATTGCGAT GGG, and GCTACCACTGGTTCTGATCG GGG for *Nr2e3* knockdown. pAAV-nEFCas9 (nEF, hybrid EF1α/HTLV) was previously published[11]. Cas9 expression plasmid (hCas9) was purchased from Addgene (41815).

T7E1 assay

To examine the efficacy of the gRNA, we performed the T7E1 assay in immortalized mouse fibroblasts. Successful knockdown induces gene mismatch sites which can be cut by T7E1 nuclease. Briefly, cells were transfected with pAAV-U6-gRNA and hCas9 (Addgene 41815) using Lipofectamine 2000 (ThermoFisher). Two days after transfection, the cells were harvested and genomic DNA was extracted with DNeasy Blood & Tissue kit (QIAGEN), which is used for T7E1 (NEB) assay following manufacturer's instructions. Primers to amplify genomic regions are listed as following: *NRL*-F: ACCTCTCTCTGCTCAGTCCC; *NRL*-R: GACATGCTGGGCTCCTGTC; *NR2E3*-F: GAAGACGAGACCAGGGCAAA; *NR2E3*-R: TCTGGTTGCAGACACAGACG.

AAV production

All AAVs were packaged with serotype 8 and were generated by the Gene Transfer Targeting and Therapeutics Core (GT3) at the Salk Institute for Biological Studies.

Animals

C57BL/6, rd10, and FVB/N mice were purchased from the Jackson laboratory. All mice used in this study were of mixed gender. All procedures were conducted with the approval and under the supervision of the Institutional Animal Care and Use Committee (IACUC) at the University of California San Diego and adhered to the ARVO Statement for the Use of Animals in Ophthalmic and Vision Research.

Subretinal injection in mouse

Normal wild type **(**C57/BL6), rd10, and FVB/N mouse were used in the study. Subretinal injection into P7 neonate eyes was performed as previously described[12, 13]. Experimental mice were anesthetized with an intraperitoneal injection of a mixture of ketamine and xylazine. Pupils were dilated with 1% topical tropicamide. Subretinal injection was performed under direct visualization using a dissecting microscope with a pump microinjection apparatus (Picospritzer III; Parker Hannifin Corporation) and a glass micropipette (internal diameter 50~75 μm). Approximately 0.5 μl AAV mixture of AAV-gRNA ($1.5\sim2\times10^{10}$GC) and AAV-Cas9 ($1\sim1.2\times10^{10}$GC) was injected into the

subretinal space through a small scleral incision. Injection site was located superior temporal, 0.5mm behind limbus margin. A successful injection was judged by creation of a small subretinal fluid bleb. Fundus examination was performed immediately following injection, and mice showing any sign of retinal damage such as bleeding were discarded and excluded from the final animal counts.

ERG Recording

To monitor the efficacy of gene knock-out in rod/cone fate switch, ERG studies were performed before the animals were sacrificed for histology (P60 for rd10 and P50 for FVB/N). Mice were deeply anesthetized as described in the surgical procedure above. Eyes were treated with 1% topical tropicamide to facilitate pupillary dilation. Each mouse was tested in a fixed state and maneuvered into position for examination within a Ganzfeld bowl (Diagnosys LLC). One active lens electrode was placed on each cornea, with a subcutaneously placed ground needle electrode positioned in the tail and the reference electrodes placed subcutaneously in the head region approximately between the two eyes. Light stimulations were delivered with a xenon lamp in a Ganzfeld bowl. The recordings were processed using software supplied by Diagnosys. Photopic ERG was performed according to a published protocol[14]. Mice were light adapted for 10 minutes at a background light of 30 cd/m^2. Cone responses were elicited by a 34 cds/m^2 flash light with a low background light of 10 cd/m^2, and signals were averaged from 50 sweeps.

Histological analysis of the mouse eye

Following ERG recordings, mice were sacrificed, and retinal cross-sections were prepared for histological evaluation of ONL preservation. Mice were euthanized with CO_2, and eyeballs were dissected out, marked with injection site and then fixed in 4% PFA. Cornea, lens, and vitreous were removed from each eye without disturbing the retina. The remaining retina containing eyecup was infiltrated with 30% sucrose and embedded in OCT compound. Horizontal frozen sections were cut on a cryostat right through injected area. Retinal cross-sections were prepared for histological evaluation by Immunofluorescence staining.

Immunofluorescence

Retinal cryosections were rinsed in PBS and blocked in 0.5% Triton X-100 in 5% BSA in PBS for 1 hour at room temperature, followed by an overnight incubation in primary antibodies at 4 degree. After three washes in PBS, sections were incubated with secondary antibody. Cell nuclei were counterstained with DAPI (49,6-diamidino-2-phenylindole). For PNA staining, sections were incubated with PNA at room

temperature after secondary antibody for one hour. The following antibodies were used: mouse anti-Rhodopsin monoclonal antibody (Abcam ab3267), rabbit anti-Opsin polyclonal antibody (Millipore AB5407), rabbit anti-Cone Arrestin polyclonal antibody (Millipore AB15282). The secondary antibodies, Alexa Fluor-488- or 555-conjugatedanti-mouse or rabbit immunoglobulin G (IgG) (Invitrogen) were used at a dilution of 1:500. Rhodamine labeled PNA (Vector RL-1072) was used at a dilution of 1:200. Sections were mounted with Fluoromount-G (Southern Biotech) and coverslipped. Images were captured by an Olympus FV1000 confocal microscope. All images were taken and measured at the same retina area position.

qRT-PCR from mouse retinas

Following ERG recordings, mice were sacrificed. RNA was isolated from retina tissue using AllPrep DNA/RNA Mini Kit (Qiagen). cDNA was synthesized from RNA using Superscript III reverse transcriptase kit (Invitrogen). Quantitative PCR was performed via 40 cycle amplification and Power SYBR Green PCR Master Mix on a 7500 Real-Time PCR System (Applied Biosystems). Measurements were performed in triplicate and normalized to endogenous *GAPDH* levels. The relative fold change in expression was calculated using the ΔΔCT method (CT values <30). Primers for qPCR are listed as following:

Gene	Forward Primer	Reverse Primer
Nrl	ccttctgagggccgatctg	gacatgctgggctcctgtc
Nr2e3	accagtcccaggtgatgcta	ctcaaagatgggagcaggag
Crx	aaactgagctgggatgctgt	ttgtgccccctcaatctaac
Rho	tcagtctgcatccctcctct	cccagtttccatccattttg
PDE6b	gccgtgtttcatggcttt	tccaaagttacattcgatcttttt
GNAT1	cccctcaaataccgtccttt	gctgctgtaggtccaagagg
Arr3	aaagctccaacagcagacaga	tggacaaaatgatttattagagtgttg
Opn1mw	tctctttggaaagaaggttgatg	tgagaagggaggtaaaacatgg
Opn1sw	cagaatggcctcacctcatt	aggagcagcaggtgtaagga
PDE6c	atccaaaagagcctccttga	tttccaggtcagcaatggat
GNAT2	aaaccaccccaaagcctaac	gaaataagcaggctcgcatc
Gapdh	cgtcccgtagacaaaatggt	ttgatggcaacaatctccac

Statistical analysis

Results are shown as mean±s.d or mean±s.e.m., as indicated in the figure legend. Comparisons were performed with student's *t*-test or paired student t-test, as indicated in the figure legend.

Supplemental References

11 Suzuki K, Tsunekawa Y, Hernandez-Benitez R *et al.* In vivo genome editing via CRISPR/Cas9 mediated homology-independent targeted integration. *Nature* 2016; **540**:144-149.

12 Matsuda T, Cepko CL. Electroporation and RNA interference in the rodent retina in vivo and in vitro. *Proceedings of the National Academy of Sciences of the United States of America* 2004; **101**:16-22.

13 Wang S, Sengel C, Emerson MM, Cepko CL. A gene regulatory network controls the binary fate decision of rod and bipolar cells in the vertebrate retina. *Developmental cell* 2014; **30**:513-527.

14 Usui S, Komeima K, Lee SY *et al.* Increased expression of catalase and superoxide dismutase 2 reduces cone cell death in retinitis pigmentosa. *Molecular therapy : the journal of the American Society of Gene Therapy* 2009; **17**:778-786
